# Supplementary material for: Passive Voice Comprehension during Thematic-Role Assignment in Russian-Speaking Children Aged 4–6 Is Reflected in the Sensitivity of ERP to Noun Inflections
Source: Brain Sci. 2022 May 27;12(6):693. doi: 10.3390/brainsci12060693 (PMC9220815; doi:10.3390/brainsci12060693)
Supplement: Supplementary file 1 [file brainsci-12-00693-s001.zip › brainsci-1671736-supplementary.pdf]

Table S1. Behavioral, psychological and speech development assessment results in 3 groups of children.

	AV Correct Answers (%)	PV Correct Answers (%)	Phonological Awareness (%)	Syntactic Awareness (%)	Nonverbal Intellect (Points, Raven's Color Progressive Matrices)	Working Memory (Items)
4 y.o.	81.1	78.1	72.8	76.1	20.4	4.4
5 y.o.	88.8	80.4	82.3	83.8	20.9	4.7
6 y.o.	96.1	92	86	90.3	26.1	4.9
